# A first report of right-hemicolectomy for ascending colon cancer in Japan with the da Vinci SP surgical robot system

**DOI:** 10.1186/s40792-024-01922-w

**Published:** 2024-05-21

**Authors:** Ai Noda, Koichi Okuya, Emi Akizuki, Masaaki Miyo, Masayuki Ishii, Ryo Miura, Momoko Ichihara, Maho Toyota, Tatsuya Ito, Tadashi Ogawa, Akina Kimura, Ichiro Takemasa

**Affiliations:** Department of Surgery, Surgical Oncology and Science, Sapporo Medical University School of Medicine, 291 S1 W16, Chuo-Ku, Sapporo, Hokkaido 060-8543 Japan

**Keywords:** Right colectomy, Minimally invasive surgery, Robotic single-incision laparoscopic surgery, Robotic surgery, Single-port colectomy, Ascending colon cancer

## Abstract

**Background:**

The da Vinci SP robotic surgical system received regulatory approval for use in colorectal cancer surgery in Japan in April 2023. Given the advantages of the precision of a robot and the postoperative cosmesis of single-site surgery, the system is expected to be further utilized for minimally invasive surgeries, in addition to the curative and safety-assured laparoscopic technique.

**Case presentation:**

A 73-year-old man presented at our hospital with positive fecal occult blood. He was diagnosed with cT2N0M0 (Stage I) ascending colon cancer and underwent a right hemicolectomy, which was performed with the da Vinci SP system. The operation was performed safely, and the patient was discharged without complications. Pathology findings showed that complete mesocolic excision was achieved.

**Conclusions:**

Herein, we report the first colorectal cancer surgery performed using the da Vinci SP system in Japan. The use of this robotic surgical system with access forms for right hemicolectomy is safe and oncologically appropriate.

## Background

Based on the results of large randomized controlled trials comparing open and laparoscopic surgeries, the latter is considered an option for colorectal cancer and is widely performed as a minimally invasive surgery [1, 2]. Compared with laparoscopic surgery, robotic surgery has shown superior short-term outcomes in rectal cancer surgery and has the potential to overcome the issues of the laparoscopic technique and provide higher quality outcomes in colon cancer surgery. In Japan, robotic surgery for rectal resection was covered by insurance in 2018, whereas that for colon cancer surgery was covered in 2022, and such coverage is expected to spread to other medical conditions with the increasing societal need for minimally invasive surgery. In Japan, a prospective cohort study on robotic surgery for colon cancer demonstrated the safety and curative potential of the surgical system [3]. The world's first minimally invasive single-incision laparoscopic surgery (SLS) was reported in 2008 [4], and many studies have shown its safety and favorable oncologic outcomes compared with those of multiport laparoscopic surgery [5–8]. The da Vinci SP system (Intuitive Surgical Inc., Sunnyvale, CA, USA) is a specialized surgical platform that enables single-port robot-assisted surgery. In Japan, it was covered by insurance in April 2023 for use in colorectal surgeries. Herein, we report the first colon cancer surgery using the da Vinci SP system in Japan.

## Case presentation

**Fig. 1:**
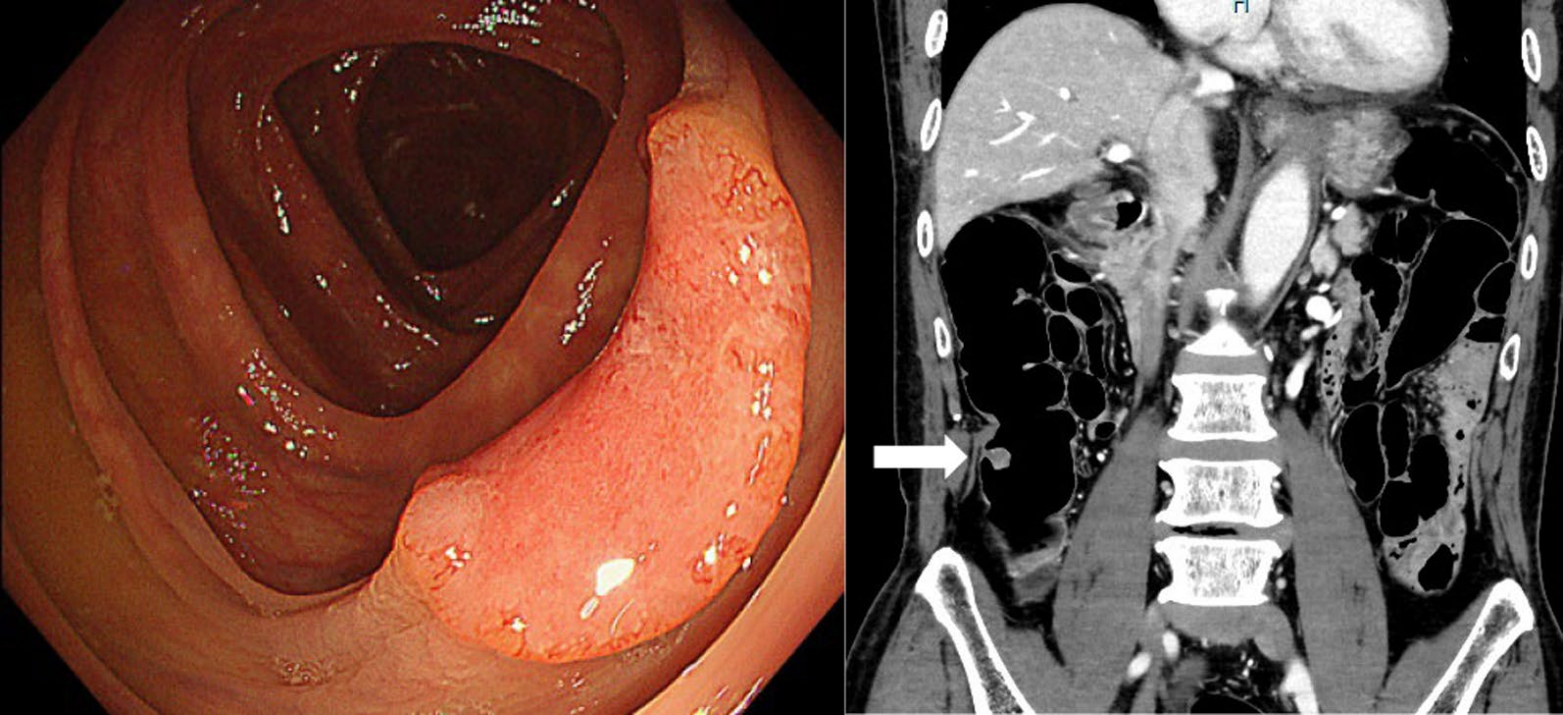
Preoperative colonoscopy examination and enhanced computed tomography (CT) scan. The colonoscopy indicates a type 2 tumor in the ascending colon, and the CT image reveals wall thickening in the ascending colon (white arrow)

**Fig. 2:**
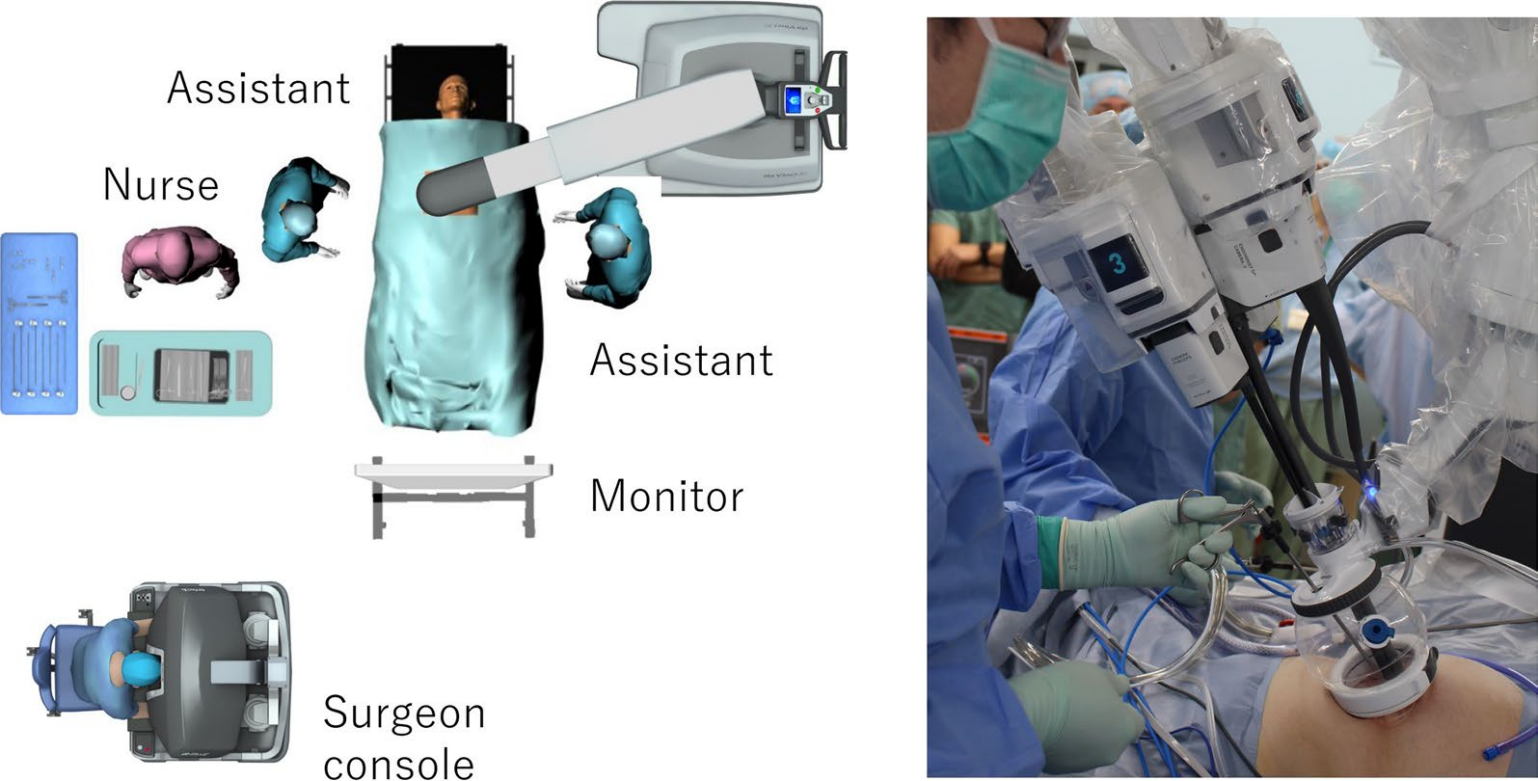
Operation setup of the da Vinci SP system. The da Vinci SP Access Port kit, large incision (dedicated da Vinci SP access form) is balloon shaped, with an access hole for use of assistant instruments

**Fig. 3:**
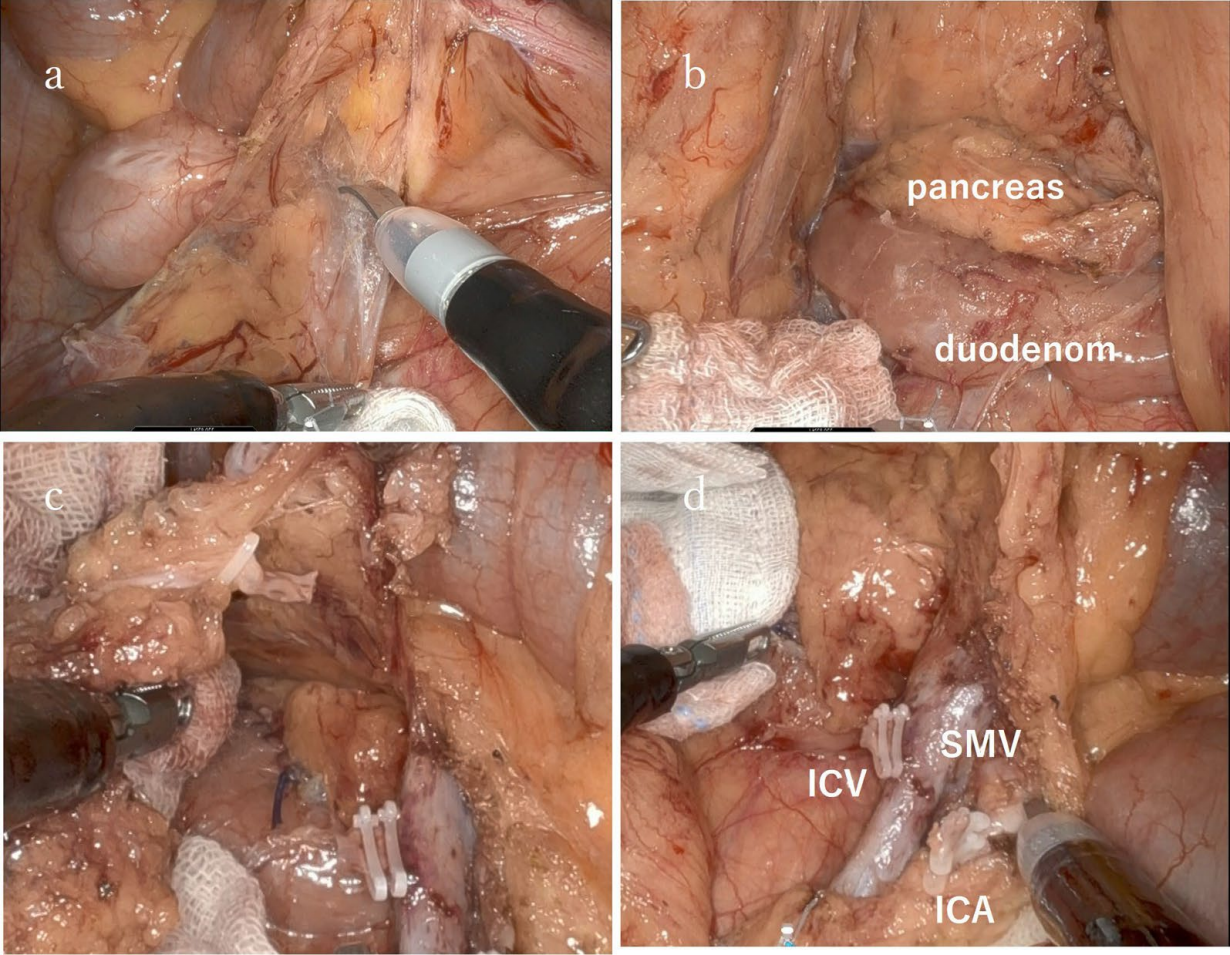
Intraoperative findings during the robotic right hemicolectomy. **a** Start of the inferior approach. **b** After the inferior approach. **c** Ligation of the ileocolic vein. **d** After lymph node dissection. *ICA* ileocolic artery, *ICV* ileocolic vein, *SMV* superior mesenteric vein

A 73-year-old man (BMI: 22.1 kg/m^2^) presented at our hospital for close examination of positive fecal occult blood. Endoscopic examination revealed a tumor in the ascending colon, and the biopsy results indicated adenocarcinoma. Computed tomography imaging showed no lymph node metastasis or distant metastasis. His cancer was diagnosed as cT2N0M0 (Stage I) (Fig. 1). A right hemicolectomy was performed using the da Vinci SP system. The surgeon had experience in more than 500 single-site laparoscopic colorectal surgeries. The use of the da Vinci SP system for the colorectal cancer surgery was approved by the Evaluating Committee for Highly Difficult New Medical Technologies (Approval number 23-001). A 3.5 cm incision was made in the umbilicus, following which a uniportal access device (Access Port kit, large incision; Intuitive Surgical Inc.) was fitted into the incision the assistant use assist port to move the gauze in and out of the surgical site (Fig. 2). First, the head of the patient was positioned 10°downward and 12°left downward. In this point, the camera was in the below position. Then, after the small intestine had been positioned to the left cranial side, the da Vinci SP system was docked to the patient over the left side. The instruments used in this surgery were fenestrated bipolar forceps for the robotic first arm, Cadiere forceps and fenestrated bipolar forceps for the second arm, and monopolar curved scissors or Maryland bipolar forceps for the third arm. The mesentery of the colon was mobilized using an inferior approach, ensuring exposure of the duodenum and pancreatic head. Subsequently, the head of the patient was positioned 10°upward, and the camera was changed to the above position. The ileocecal vessels were clipped and dissected, and complete mesocolic excision (CME) with central vascular ligation was executed. The right-sided colon was extracted through the umbilical site after dissection of the greater omentum and mobilization of the hepatic flexure. Because the da Vinci SP system does not have a stapling device, extracorporeal functional end-to-end anastomosis was performed (Fig. 3).

**Fig. 4:**
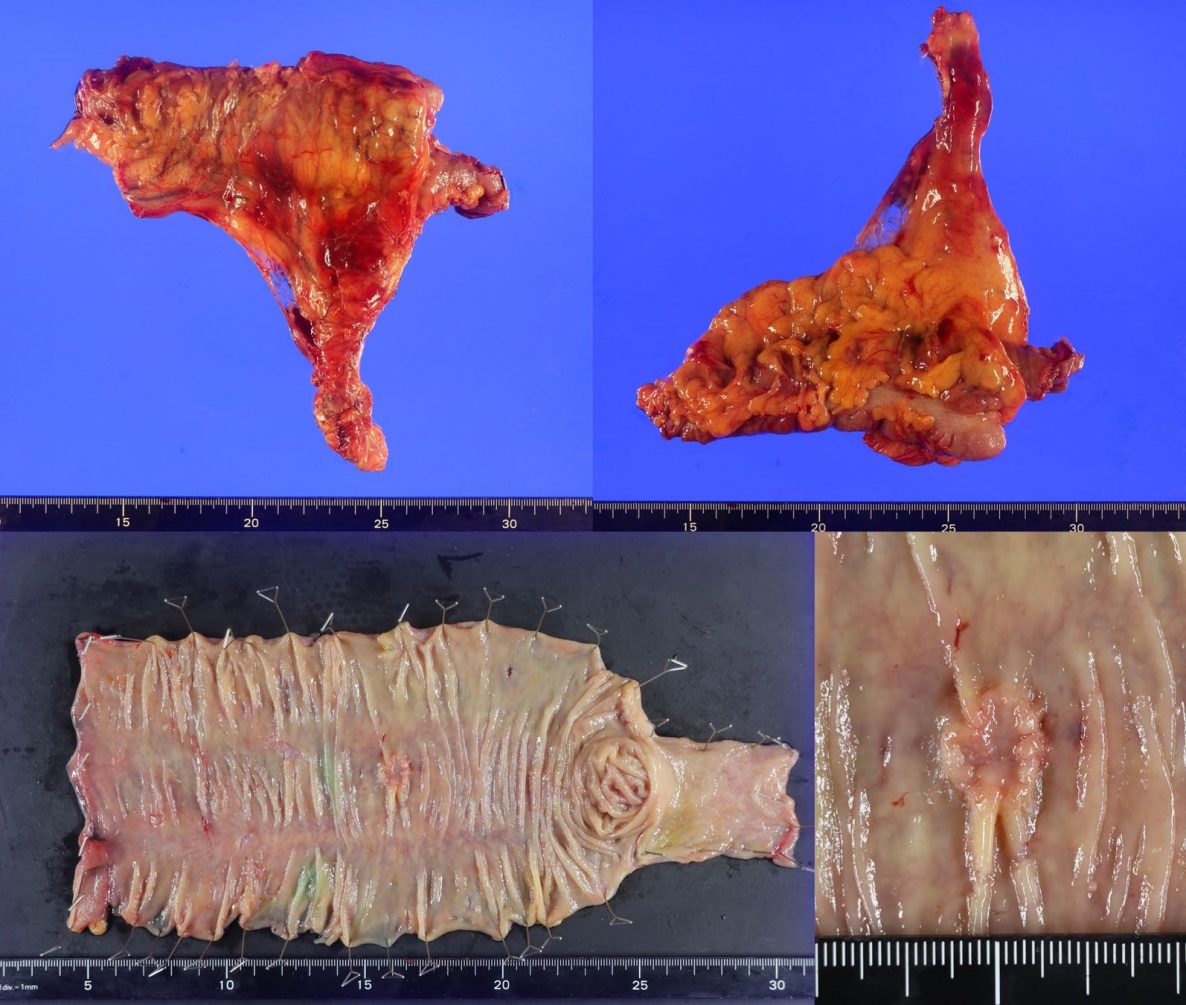
Pathological specimen

**Fig. 5:**
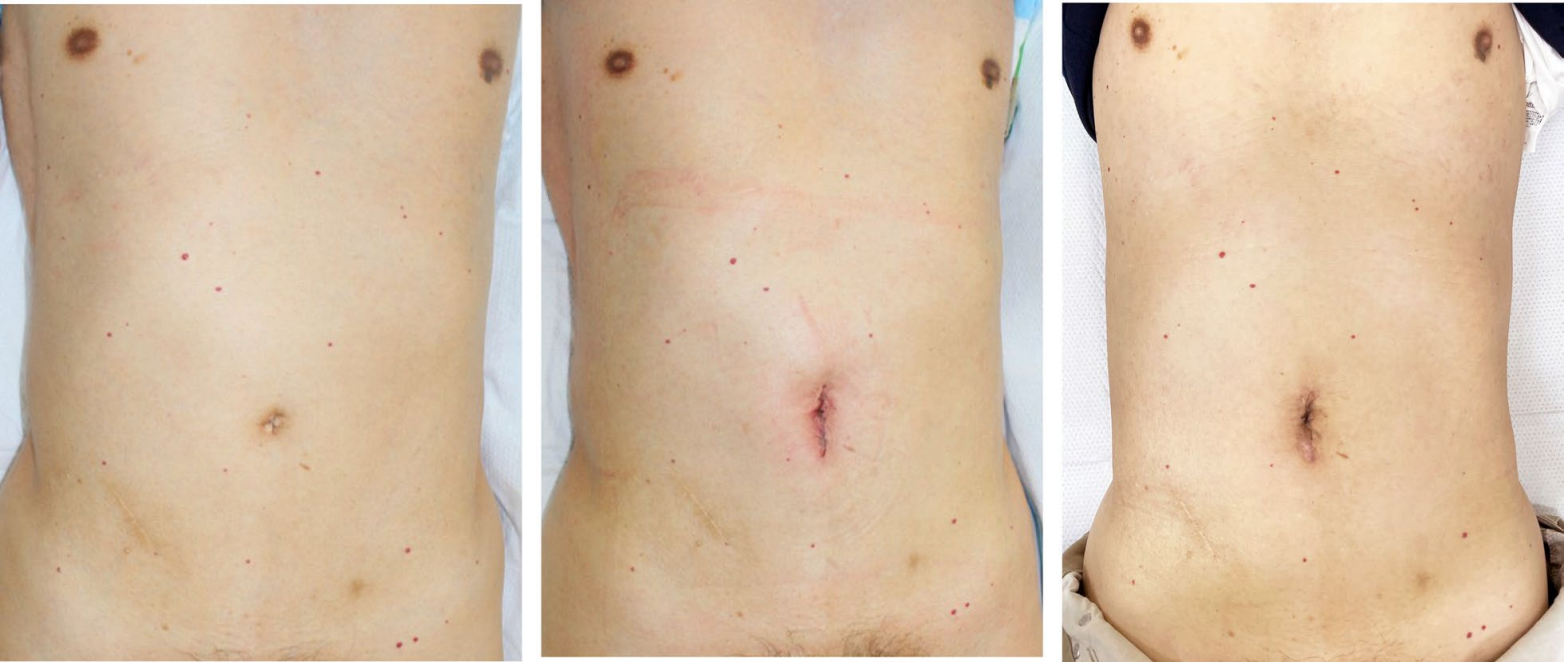
Surgical wound site before and after the procedure. Surgical wound site before, immediately after, and 2 weeks after the procedure

The procedure required one docking with a docking time of 20 min. The total operative time was 239 min, the console time was 147 min, and the blood loss was 5 mL. The patient was discharged on postoperative day 8 without complications. The pathological result was pT2N0M0 (Stage I), as diagnosed preoperatively (Fig. 4). Figure 5 shows the surgical wound site before and after the procedure.

## Discussion

Currently, we are in an era where the integration of robotic surgery (which pursues precision) and single-port surgery (which pursues cosmesis) is required. Although several cases involving robotic single-incision laparoscopic surgery (rSLS) have been reported, the number is low, because it is not easy for a bulky multi-arm robot to operate from a single incision site [9–11]. The da Vinci SP system was developed for rSLS. This robot, like the da Vinci Xi system, is the fourth generation of the da Vinci brand, and although it is comparable in operation, some differences exist.

**Fig. 6:**
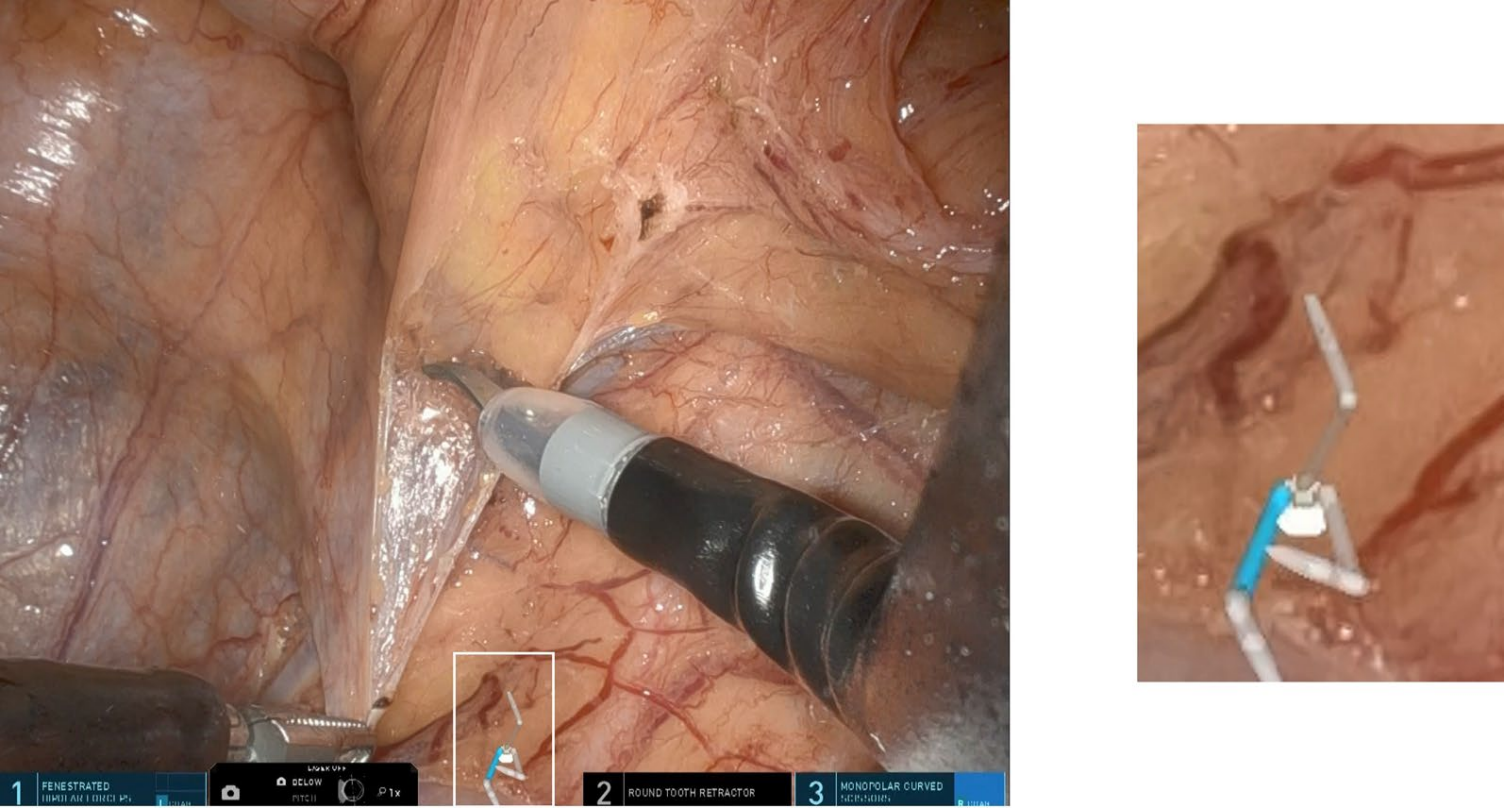
Articulated function of forceps and the holographic navigation system

When performing a right hemicolectomy, wide separation between the anterior surface of the pancreas and the ascending mesentery is considered important to avoid anatomical misidentification. We emphasize this point in the inferior approach, which reduces the risk of pancreatic injury. In multiport laparoscopic colectomy (MLC), forceps are used to create a large triangle by traction from outside the body for dissection of the mesocolic fascia and retroperitoneum. In conventional SLS, the forceps are pulled from inside of the body to the outside to form a triangulation system, which limits the distance they can be pulled compared with that of MLC, making the creation of a large surface difficult. However, with the da Vinci SP system, each pair of forceps is bent to allow sufficient traction from the inside to the outside. In addition, accessibility is increased because of the umbilical port site created. As a result, although the triangulation system is smaller than that in MLC, it can be continuously created while moving, facilitating a smooth CME process. Conventionally, SLS uses two pairs of forceps in addition to the camera. By contrast, the da Vinci SP system allows the use of three pairs of forceps in addition to the camera, which is a great advantage in surgical field formation. For example, when creating a triangulation system with the surgical trunk as the base, it is possible to use two more pairs of forceps in addition to arm 2, which holds the apex of triangulation, allowing a better field of view. Moreover, since all forceps are inserted in parallel and the tips can then be bent inward from the outside, there is no conflict between the instruments (Fig. 6). With regard to confliction, the holographic navigation system on the console screen allows the surgeon operating the da Vinci SP system to specifically track the spatial positioning of the forceps, minimizing the interference of an extra pair of forceps (compared with SLS) and allowing for a more efficient use of space. This is an extremely useful new feature in the integration of robotic and single-port surgeries. The da Vinci SP system is equipped with a flexible camera that can be bent in the same way as the forceps. This means that the cobra position can be easily manipulated as needed, which is very useful for avoiding an in-line view by creating a bird's eye view and also greatly reduces the limitation of surgical field development due to forceps interference. Although articulated forceps are very useful with the da Vinci SP system, an effective length of 10 cm from the port site to the operative field is required to obtain flexion. Therefore, even though this is unlikely to be a problem for handling in the pelvis, an access port needs to be devised for manipulation close to the umbilicus. The use of GelPOINT (Applied Medical, Inc., Rancho Santa Margarita, CA, USA) for colon resection has been reported [11], but this point has largely been resolved because a dedicated balloon access form (Fig. 2) has been developed. However, unlike earlier models of the robot, the da Vinci SP system has no stapler and sealing device. Although assistants can operate these devices from the Access Port kit, they are not very maneuverable. If these devices are introduced, it may be possible to operate them more conveniently. Improvements in the near future are expected.

Based on what has been discussed so far, surgery with the da Vinci SP system will be a relatively smooth transition for the surgeons that already have both robotic and SLS techniques. In particular, the availability of the holographic navigation system and special camera positions allows for an easier and more objective grasp of the intraperitoneal environment, which greatly facilitates the operation. In other words, this is a new and remarkable system that eliminates the individual disadvantages of robotics and SLS and combines their advantages in their integration.

## Conclusions

The right hemicolectomy using the da Vinci SP system with dedicated access forms was safe to perform in our patient. Moreover, CME could be successfully accomplished and was considered oncologically appropriate.

## Data Availability

Not applicable.

## Abbreviations

SLS: Single-incision laparoscopic surgery
CME: Complete mesocolic excision
rSLS: Robotic single-incision laparoscopic surgery
MLC: Multiport laparoscopic colectomy
